# Supplementary material for: Giant antidamping orbital torque originating from the orbital Rashba-Edelstein effect in ferromagnetic heterostructures
Source: Nat Commun. 2018 Jul 2;9:2569. doi: 10.1038/s41467-018-05057-z (PMC6028484; doi:10.1038/s41467-018-05057-z)
Supplement: Supplementary file 1 — Supplementary Information [file 41467_2018_5057_MOESM1_ESM.docx]

Supplementary Information for

**Giant antidamping orbital torque originating from the orbital Rashba-Edelstein effect in ferromagnetic heterostructures**

by Chen et al.

Supplementary Note 1: Derivation of Eqs. 2 and 3 of the main text

To quantify the current-induced torque, we used a macrospin method to model the torque interplay as shown in Supplementary Figure 1. The slightly tilt of the external magnetic field **H**^ext^ away from the *xy*-plane with angle *β* was introduced to prevent domain formation, such that the magnetization can rotate coherently and the macrospin method is applicable. In a quasistatic **H**^ext^ sweeping process, the equilibrium orientation of the magnetization vector **m**, *i.e.*, *θ*, which is the angle between **m** and the film normal **z**, can be determined from the condition that the magnitude of the total torque *T*^tot^ on **m** is zero^1^, i.e.,

$T^{\text{tot}}=x\cdot(T^{\text{AD}}+T^{\text{ext}}+T^{\text{an}})=0$ (1)

where **T**^AD^ is the current-induced antidamping torque, **T**^ext^ and **T**^an^ are the torques from **H**^ext^ and the anisotropy field **H**^an^, respectively. The current-induced field-like torque **T**^FL^ has been neglected here according to the results of Figure 3b of the main text, which shows that the **T**^FL^ is negligibly small. The magnitude of the current-induced Oersted field was calculated to be about 0.45 Oe for a current magnitude of 0.9 mA in our Pt (5)/ Co(1)/SiO_2_ (1)/Pt (1) (nm) devices based on Ampere's law, which is considerably smaller than the magnitude of **T**^AD^ (i.e., *T*^AD^) and has also been neglected. When considering the space symmetry of torques^2^, **T**^AD^ has the form **T**^AD^ = **m** × (**σ** × **m**) *T*AD 0 + (**z** × **m**)(**m**·**y**)[*T*AD 2 + *T*AD 4(**z** × **m**)^2^] = (*T*AD 0 + *T*AD 2sin^2^*θ* + *T*AD 4sin^4^*θ*)**x** = *T*^AD^**x**, and its corresponding effective field **H**^AD^ takes the form **H**^AD^ = (*H*AD 0 + *H*AD 2sin^2^*θ* + *H*AD 4sin^4^*θ*)(**m** × **σ**) = *H*^AD^(**m** × **σ**), when the current is applied along **y** and **m** rotates in the *yz*-plane. Here, *T*AD *n* and *H*AD *n* (*n* = 0, 1, and 2) correspond to the zeroth-, second-, and fourth-order terms in *T*^AD^ and *H*^AD^, respectively, and **σ** is the unit vector of the current-induced nonequilibrium orbital polarization Δ**L** generated by the orbital Rashba-Edelstein effect (see Figure 6c of the main text). It is straightforward to have

$T^{\text{AD}}=T_{0}^{\text{AD}}+T_{2}^{\text{AD}}\sin^{2}\theta+T_{4}^{\text{AD}}\sin^{4}\theta$ (2)

and $H^{\text{AD}}=H_{0}^{\text{AD}}+H_{2}^{\text{AD}}\sin^{2}\theta+H_{4}^{\text{AD}}\sin^{4}\theta$. (3)

It is obvious that **T**^AD^ is equal in magnitude to **H**^AD^, *i.e.*, *T*^AD^ = *H*^AD^.

The magnetic energy *E* of the Pt/Co/SiO_2_/Pt heterostructures with uniaxial perpendicular magnetic anisotropy (PMA) can be expressed as

$E=K_{\text{u}}-M_{\text{s}}m\cdot(H^{\text{ext}}+H^{\text{AD}})$, (4)

where *K*_u_ = *K*_1_sin^2^*θ* + *K*_2_sin^4^*θ* + *K*_3_sin^6^*θ* is the uniaxial magnetic anisotropy energy and *M*_s_ is the saturation magnetization. *K*_1_, *K*_2_, and *K*_3_ are the anisotropy constants. It is sufficient to consider only the *K*_1_, *K*_2_, and *K*_3_ terms in this study. The product of *M*_s_**m**⋅**H**^AD^ is equal to zero because **m** is always orthogonal to **H**^AD^ according to the torque symmetry. Then, Supplementary Equation 4 is changed to

$E=K_{1}\sin^{2}\theta+K_{2}\sin^{4}\theta+K_{3}\sin^{6}\theta-M_{\text{s}}H^{\text{ext}}\cos(\text{π}/2-\theta)$, (5)

At the magnetization equilibrium condition δ*E*(*θ*)/δ*θ* = 0, we have

$(\frac{2K_{1}}{M_{s}}+\frac{4K_{2}}{M_{s}}\sin^{2}\theta+\frac{6K_{3}}{M_{s}}\sin^{4}\theta)\sin\theta-H^{\text{ext}}=0$. (6)

Here, we define the magnitude of **H**^an^, i.e., *H*^an^, as

$H^{\text{an}}=\frac{2K_{1}}{M_{s}}+\frac{4K_{2}}{M_{s}}\sin^{2}\theta+\frac{6K_{3}}{M_{s}}\sin^{4}\theta=H_{0}^{\text{an}}+H_{2}^{\text{an}}\sin^{2}\theta+H_{4}^{\text{an}}\sin^{4}\theta$, (7)

where *H*an 0 = 2*K*_1_/*M*_s_, *H*an 2 = 4*K*_2_/*M*_s_, and *H*an 4 = 6*K*_3_/*M*_s_. By substituting Supplementary Equations 2 and 7 into Supplementary Equation 1, we obtain

$\sum_{n=0,2,4} T_{n}^{\text{AD}}\sin^{n}\theta+H^{\text{ext}}\cos(\theta+\beta)-(\sum_{n=0,2,4} H_{n}^{\text{an}}\sin^{n}\theta)\cos\theta\sin\theta=0$, (8)

where *H*^ext^ is the magnitude of **H**^ext^. We define *H*ext +(*θ*) and *H*ext – (*θ*) as the *H*^ext^ values that produce the same *θ* for positive (along **y**) and negative (along −**y**) currents, respectively. Then, we have

$\sum_{n=0,2,4} T_{n}^{\text{AD}}\sin^{n}\theta+H_{\text{+}}^{\text{ext}}(\theta)\cos(\theta+\beta)-(\sum_{n=0,2,4} H_{n}^{\text{an}}\sin^{n}\theta)\cos\theta\sin\theta=0$, (9)

$-\sum_{n=0,2,4} T_{n}^{\text{AD}}\sin^{n}\theta+H_{-}^{\text{ext}}(\theta)\cos(\theta+\beta)-(\sum_{n=0,2,4} H_{n}^{\text{an}}\sin^{n}\theta)\cos\theta\sin\theta=0$. (10)

By solving the simultaneous equations using a combination of Supplementary Equations 9 and 10, we obtain

$H_{-}^{\text{ext}}(\theta)-H_{+}^{\text{ext}}(\theta)=2(T_{0}^{\text{AD}}+T_{2}^{\text{AD}}\sin^{2}\theta+T_{4}^{\text{AD}}\sin^{4}\theta)/\cos(\theta+\beta)$, (11)

$H_{-}^{\text{ext}}(\theta)+H_{+}^{\text{ext}}(\theta)=2(H_{0}^{\text{an}}+H_{2}^{\text{an}}\sin^{2}\theta+H_{4}^{an}\sin^{4}\theta)\cos\theta\sin\theta/\cos(\theta+\beta)$. (12)

When *T*AD *n* = *H*an *n* = 0 (*n* = 2 and 4), Supplementary Equations 11 and 12 simplify to the case in Supplementary Reference 1, where only the zeroth-order terms *T*AD 0 and *H*an 0 was considered. Note that *β* is fixed at 3°, and cos*θ* and sin*θ* can be accurately determined based on the normalized anomalous Hall effect (AHE) curves shown in Figure 3a of the main text. Therefore, by taking the difference between *H*ext +(*θ*) and *H*ext –(*θ*) or sum of *H*ext +(*θ*) and *H*ext –(*θ*), the values of *T*AD *n* and *H*an *n* (*n* = 0, 2, and 4) can be obtained by performing fits using Supplementary Equations 11 and 12, respectively.


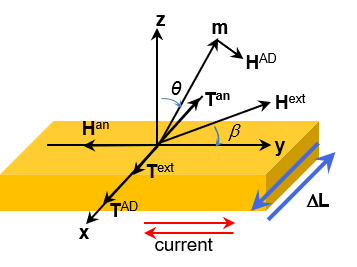


**Supplementary Figure 1 | Measurement and modelling geometry**. Illustration of the interplay between **T**^AD^, **H**^ext^, **H**^an^. **H**^ext^ and **m** are in the *yz*-plane. Δ**L** is the current-induced nonequilibrium orbital polarization generated by the orbital Rashba−Edelstein effect. **H**^AD^ is the effective field corresponding to **T**^AD^. *θ* is the angle between **m** and the normal direction **z** and *β* is the angle between **H**^ext^ and the *xy*-plane.

Supplementary Note 2: Separation of AHE and planar Hall effect (PHE)

Supplementary Figure 2a shows a top view of a Pt (5)/Co (1)/SiO_2_ (1)/Pt (1) (nm) device. When a charge current *I* is supplied to the device, a longitudinal voltage *V*_yy_ and a transverse voltage *V*_xy_ can be detected. *V*_xy_ contains AHE and planar Hall effect (PHE) signals. The total Hall resistance *R*_H_ can be obtained according to Ohm’s law *R*_H_ = *V*_xy_/*I*. In principle, PHE has no contribution to *R*_H_ when **H**^ext^ rotaes strictly within the *yz*- or *xz*-plane. In this case, *R*_H_ purely comes from the AHE resistance (*R*_AHE_). However, an unintentional tilting of **H**^ext^ from the *yz*- or *xz*-plane creates a magnetization component within the *xy*-plane, causing the PHE. It is necessary to separate the *R*_AHE_ and PHE resistance (*R*_PHE_) contributions because the determination of *θ* in Supplementary Equations 11 and 12 is based on the AHE only. The separation of *R*_PHE_ and *R*_AHE_ can be achieved by measuring *R*_H_ as a function of *H*^ext^. Supplementary Figure 2b shows the *R*_H_ vs. *H*^ext^ curve of a Pt (5)/Co (1)/SiO_2_ (1)/Pt (1) (nm) device, measured with a current of 0.9 mA and *β* = 3°. As the AHE and PHE are respectively odd and even with respect to **m**, the *R*_AHE_ and *R*_PHE_ contributions can be separated by the antisymmetrization and symmetrization of the *R*_H_, respectively. This is achieved by inverting *R*_H_ (+*H*^ext^ → −*H*^ext^ and −*H*^ext^ → +*H*^ext^) with respect to the origin of the curve and taking the sum or difference with *R*_H_ (−*H*^ext^ → +*H*^ext^ and +*H*^ext^ → −*H*^ext^), as shown in Supplementary Figures 2c and 2d. It is seen that the *R*_PHE_ is two orders of magnitude smaller than the *R*_AHE_, and thus have negligible effect on our experiment.


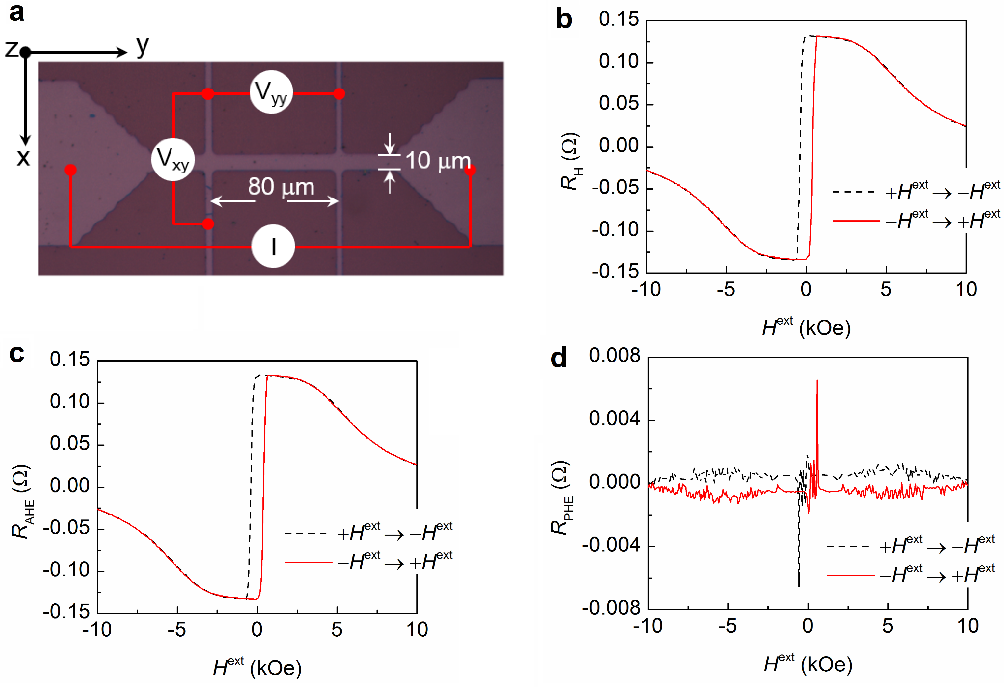


**Supplementary Figure 2 | Separation of AHE and PHE**. **a** Top view of a Pt (5)/Co (1)/SiO_2_ (1)/Pt (1) (nm) device. The electric current *I* is injected along the *y*-axis. *V*_yy_ is the longitudinal voltage. *V*_xy_ is the transverse voltage that contains AHE and PHE signals. **b** Total Hall resistance *R*_H_ as a function of external magnetic field *H*^ext^, measured at *β* = 3° and a current magnitude of 0.9 mA. **c** Antisymmetric AHE signal, *R*_AHE_. **d** Symmetric PHE signal, *R*_PHE_.

Supplementary Note 3: Determination of the effective Co layer thickness

Supplementary Figure 3 displays the area magnetization *M*_a_ as a function of the nominal Co layer thickness *t*_Co_ measured in Pt (5)/Co (*t*_Co_)/SiO_2_ (1)/Pt (1) (nm) samples. The samples were annealed at 200 °C for 30 minutes and measured at 300 K using vibrating sample magnetometer. The magnetic dead layer thickness *t*_DL_ is considered as the intersection between the linear fit of the raw data and the thickness axis, as indicated by the arrow. The presence of the magnetic dead layer is caused by atom interdiffusion at the Pt/Co interface^3,4^ and/or the formation of Co–O bonds at the Co/oxide interface (Figure 1c of the main text). The effective Co layer thickness (*t*eff Co) is the difference between *t*_Co_ and *t*_DL_, i.e., *t*eff Co = *t*_Co_ − *t*_DL_. For a Co layer with *t*_Co_ = 1 nm, *t*eff Co = 0.72 nm.


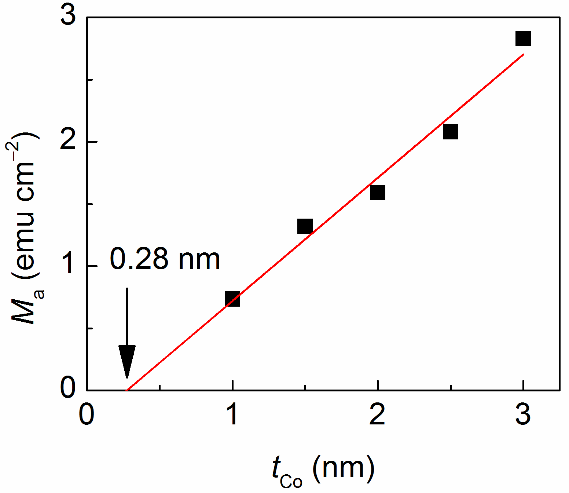


**Supplementary Figure 3 | Determination of the magnetic dead layer**. Area magnetization *M*_a_ as a function of the nominal Co layer thickness *t*_Co_ in Pt (5)/Co (*t*_Co_)/SiO_2_ (1)/Pt (1) (nm) samples. The arrow indicates the magnetic dead layer thickness.

Supplementary Note 4: Determination of the layer resistivity and current shunting

Two Pt films with thicknesses of 5 and 1 nm were deposited separately and fabricated into Hall devices. The resistivity of the 5-nm-thick and 1-nm-thick Pt layers were determined to be *ρ*_Pt5_ = 55.6 × 10^–8^ and *ρ*_Pt1_ = 80.1 × 10^–8^ Ω m at 300 K, respectively. Supplementary Figure 4 shows the equivalent circuit of a Pt (5)/Co (1)/SiO_2_ (1)/Pt (1) (nm) devices. Only conducting Pt and Co layers were considered. This is a parallel circuit and we have the equation

$\frac{1}{R_{\text{yy}}}=(\frac{t_{\text{Pt5}}}{\rho_{\text{Pt5}}}+\frac{t_{\text{Co1}}}{\rho_{\text{Co1}}}+\frac{t_{\text{Pt1}}}{\rho_{\text{Pt1}}})\frac{w}{l}$, (13)

where *R*_yy_ = *V*_yy_/*I*= 621.1 Ω is the resistance between the two Hall probes at 300 K and the channel width *w* = 10 μm and the length between the two Hall probes *l* = 80 μm. Using Supplementary Equation 13, the resistivity of the 1-nm-thick Co layer *ρ*_Co1_ was estimated to be 37.6 × 10^–8^ Ω m at 300 K. Using the equation *S* = *R*_yy_/(*ρ l*/*wt*), the current shunting percentage in the Pt (5), Co (1), and Pt (1) layers were estimated to be *S*_Pt5_ = 70%, *S*_Co1_ = 20%, and *S*_Pt1_ = 10%, respectively. We assume that the current density was uniform throughout the Pt (5)/Co (1) (nm) bilayer for the purpose of convenience. In this case, for *I* = 0.9 mA, the current density shunting in the Pt (5)/Co (1) (nm) bilayer was calculated to be *j*_PtCo_ = (*S*_Pt5_ + *S*_Co1_)*I*/(*w*(*t*_Pt5_ + *t*_Co1_)) = 1.35 × 10^6^ A cm^–2^ at 300 K.


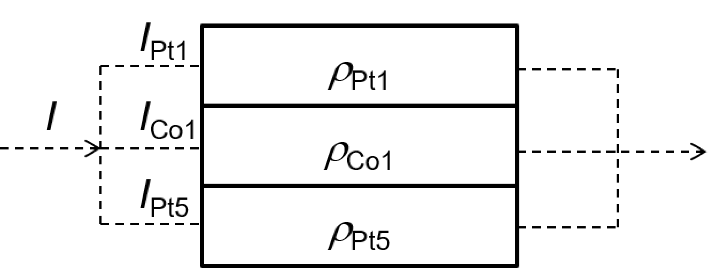


**Supplementary Figure 4 | Equivalent circuit of films**. The films have the structures of Pt (5)/Co (1)/SiO_2_ (1)/Pt (1) (in nm).

Supplementary Note 5: Joule-heating effect

To study the Joule-heating effect, we have investigated the temperature- and current-dependent *R*_yy_ of a Pt (5)/Co (1)/SiO_2_ (nm)/Pt (1) (nm) device. The device was placed in a physical property measurement system chamber. During the temperature-dependence measurement, a small *I* with magnitude of 0.1 mA was applied to excite the Hall effect while minimize the Joule heating. For the current-dependence measurement, the applied *I* started at 0.1 mA with chamber temperature of 300.1 K. It is seen from Supplementary Figure 5a that *R*_yy_ shows a linear increase with increasing temperature. Supplementary Figure 5b presents the *I* dependence of *R*_yy_, showing that *R*_yy_ also increases with increasing *I*. The linear relation between *R*_yy_ and *I*^2^ shown in the inset of Supplementary Fig. 5b indicates that the increase of *R*_yy_ is mainly caused by Joule heating. Comparison of the two figures suggests that a 0.9-mA current injection causes a negligibly small temperature increase.


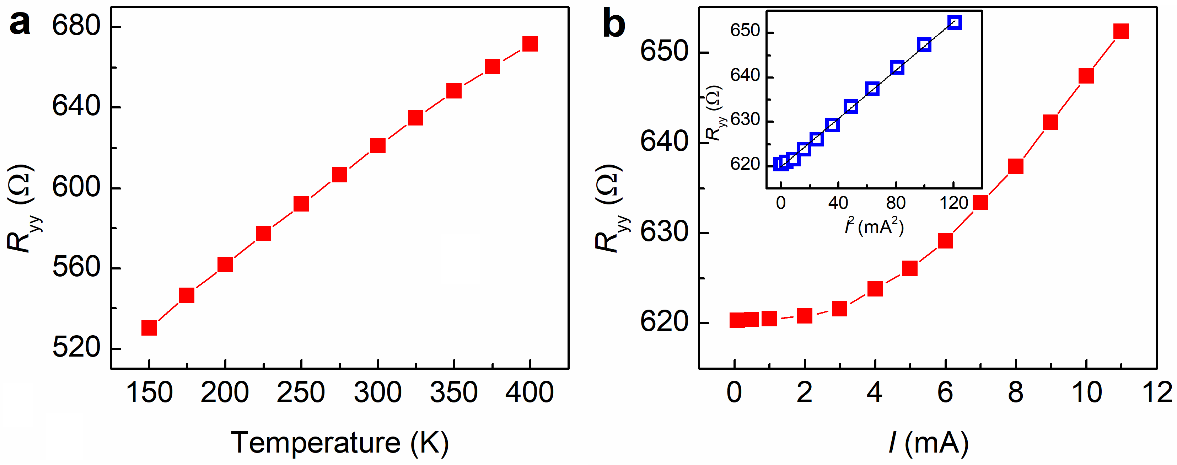


**Supplementary Figure 5 | Current-induced heating effect**. **a** temperature and **b** current (*I*) dependences of *R*_yy_ of a Pt/Co/SiO_2_/Pt device. Inset of **b**: the variation of *R*_yy_ as a function of *I*^2^.

Supplementary Note 6: Initial *θ* values in the CIMS process

Supplementary Figure 6a shows the normalized anomalous Hall effect (i.e., cos*θ*) as a function of *H*^ext^, measured in a Pt/Co/SiO_2_/Pt device with *β* = 3° and temperature of 300 K. The charge current with magnitude of 0.1 mA was applied along **y**. Supplementary Fig. 6b presents a cos*θ* vs. *H*^ext^ curve extracted from the first quadrant of Supplementary Fig. 6a. It is seen that cos*θ* decreases slowly with increasing *H*^ext^, indicating that the magnetization rotates coherently. So, we can use it to estimate the *θ* value through an inverse trigonometric transformation. The transformation result is shown in Supplementary Figure 6c. The *θ* value increases with increasing *H*^ext^. We find that the application of *H*^ext^ = 25, 50, 100, 150, 200, 250, 300, 350, and 400 Oe tilt the magnetization by approximately 0.5°, 0.7°, 1°, 1.3°, 1.7°, 2°, 2.5°, 3°, and 3.5°, which can be regarded as the initial *θ* values in the CIMS process in Figure 5 of the main text.


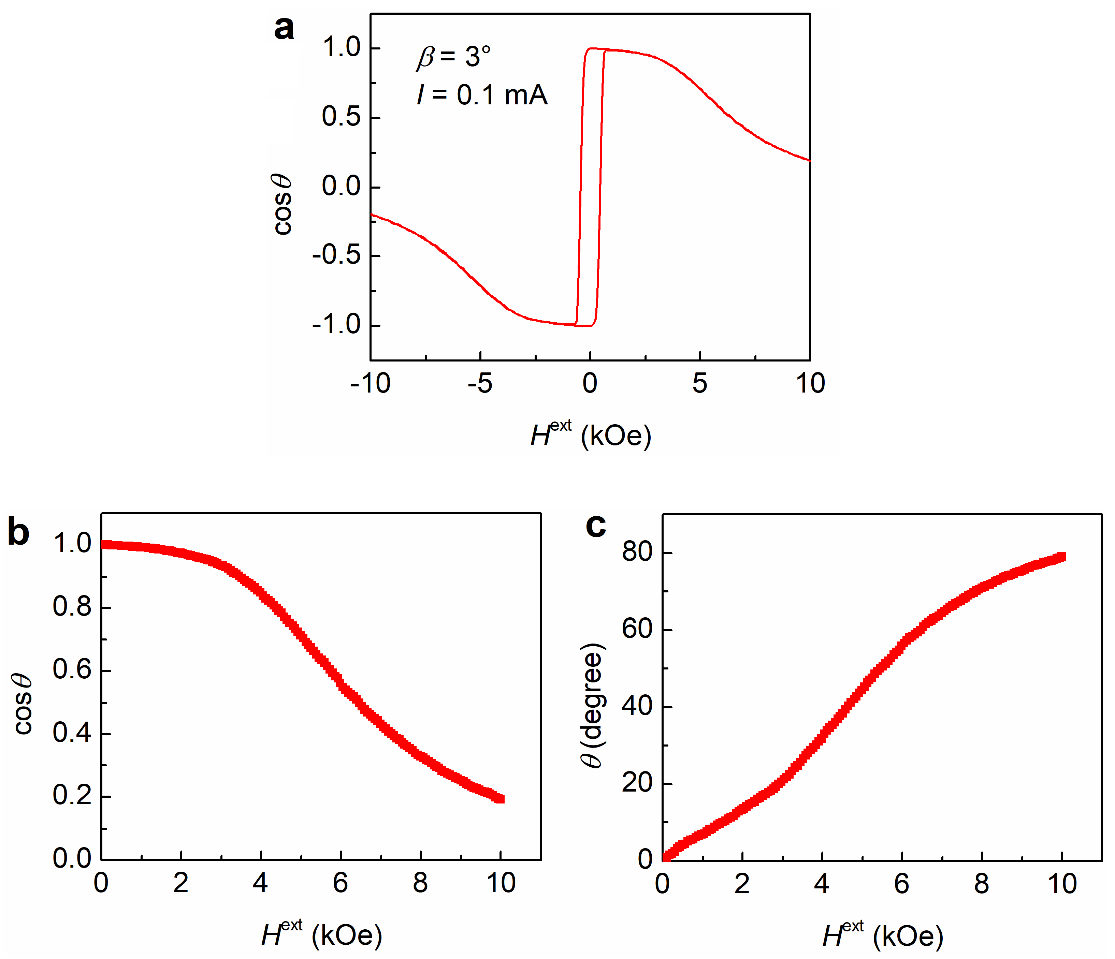


**Supplementary Figure 6 | Determination of the initial *θ* values in the CIMS process**. **a** Normalized AHE (i.e., cos*θ*) of a Pt/Co/SiO_2_/Pt device measured at 300 K. The *H*^ext^ is applied at *β* = 3° and the injected current magnitude is 0.1 mA. **b** cos*θ* vs. *H*^ext^ curve extracted from the first quadrant of **a**. **c** Variation of *θ* as a function of *H*^ext^ calculated from **b**.

Supplementary Note 7: Comments on previous experiments where the current-induced antidamping torque was found to be angular-independent

The result of the strongly angular-dependent *T*^AD^ in our Pt/Co/SiO_2_/Pt films is remarkably different from previously reported cases, for example, for example, in Pt/Co/AlO_x_ by Liu *et al*^1^, where the *T*^AD^ was showed to not have angular-dependent behavior. In our opinion, this diversity comes from the difference in the electronic structures of films. In the Pt/Co/AlO_x_, a metallic Al capping layer was first deposited on Co and subsequently oxidized into AlO_x_ by exposure to the atmosphere. In our Pt/Co/SiO_2_/Pt, the SiO_2_ layer was directly deposited from a SiO_2_ target using RF sputtering. After preparation, the Pt/Co/AlO_x_ was annealed at 350 ºC for 1 hour while in our study, the Pt/Co/SiO_2_/Pt was annealed at 200 ºC for 30 minutes. In our work, the X-ray photoelectron spectroscopy analyses indicates the presence of Co 3*d*-O 2*p* hybridization at the Co/SiO_2_ interface (Figure 1c of the main text). Although there is no such interface electronic structure characterization in the Pt/Co/AlO_x_, we speculate that it should be very different from the case in our Pt/Co/SiO_2_/Pt, because the orbital hybridization at the FM/oxide interface is sensitive to the film preparation condition, the oxide layer used, and the annealing treatment^5^. Additionally, the Pt and Co layer thicknesses in the Pt/Co/AlO_x_ are 2 and 0.6 nm, respectively, while in our Pt/Co/SiO_2_/Pt, they are 5 and 1 nm, respectively. The difference in the layer thickness can also affect the orbital hybridization at the Pt/Co and Co/(SiO_2_ or AlO_x_) interfaces^6^.

Our speculation is supported by the difference in the magnetic properties between the Pt/Co/SiO_2_/Pt and Pt/Co/AlO_x_. In the Pt/Co/AlO_x_, the authors of Supplementary Reference 1 showed that their data can be well fitted within a macrospin method where only the isotropic term *H*an 0 was considered (see Figure 2d in the Supplementary Reference 1). Since the interface orbital hybridization governs the magnetic properties, this indicates that the high-order effect of the orbital hybridization on the magnetic anisotropy is weak in the Pt/Co/AlO_x_. Correlatively, the authors of Supplementary Reference 1 found that data of the current-induce torque can be fitted when considering only the isotropic terms *T*AD 0. By contrast, we found in our study that the high-order uniaxial terms *H*an 2 and *H*an 4 in *H*^an^ are required to account for our observations (see the fitting in Figure 3d of the main text). This is also the case in *T*^AD^ (see the fitting in Figure 3c of the main text), where we found that the high-order terms *T*AD 2 and *T*AD 4 in *T*^AD^ must have to be included. These indicates that the high-order terms of the orbital hybridization have strong effects on our Pt/Co/SiO_2_/Pt films.

Supplementary References

1. Liu, L., Lee, O. J., Gudmundsen, T. J., Ralph, D. C. & Buhrman, R. A. Current-induced switching of perpendicularly magnetized magnetic layers using spin torque from the spin Hall effect. *Phys. Rev. Lett*. 109, 096602 (2012).
2. Garello, K. et al. Symmetry and magnitude of spin–orbit torques in ferromagnetic heterostructures. *Nature Nanotech.* 8, 587–593 (2013).
3. Jang, S. Y., You, C.-Y., Lim, S. H. & Lee, S. R. Annealing effects on the magnetic dead layer and saturation magnetization in unit structures relevant to a synthetic ferrimagnetic free structure. *J. Appl. Phys.* 109, 013901 (2011).
4. Bandiera, S., Sousa, R. C., Rodmacq, B. & Dieny, B. Enhancement of perpendicular magnetic anisotropy through reduction of Co-Pt interdiffusion in (Co/Pt) multilayers. *Appl*. *Phys*. *Lett*. 100, 142410 (2012).
5. Dieny, B. & Chshiev, M. Perpendicular magnetic anisotropy at transition metal/oxide interfaces and applications. *Rev*. *Mod*. *Phys*. 89, 025008 (2017).
6. Nakajima, N. et al. Perpendicular magnetic anisotropy caused by interfacial hybridization via enhanced orbital moment in Co/Pt multilayers: Magnetic circular X-ray dichroism study. *Phys*. *Rev*. *Lett*. 81, 5229 (1998).
